# Evidence For Selective Adaptation and Recalibration in the Perception of Lexical Stress

**DOI:** 10.1177/00238309211030307

**Published:** 2021-07-06

**Authors:** Hans Rutger Bosker

**Affiliations:** Max Planck Institute for Psycholinguistics, The Netherlands; Radboud University, The Netherlands

**Keywords:** Lexical stress, recalibration, selective adaptation, suprasegmental cues, prosody

## Abstract

Individuals vary in how they produce speech. This variability affects both the segments (vowels and consonants) and the suprasegmental properties of their speech (prosody). Previous literature has demonstrated that listeners can adapt to variability in how different talkers pronounce the segments of speech. This study shows that listeners can also adapt to variability in how talkers produce *lexical stress*. Experiment 1 demonstrates a *selective adaptation* effect in lexical stress perception: repeatedly hearing Dutch trochaic words biased perception of a subsequent lexical stress continuum towards more iamb responses. Experiment 2 demonstrates a *recalibration* effect in lexical stress perception: when ambiguous suprasegmental cues to lexical stress were disambiguated by lexical orthographic context as signaling a trochaic word in an exposure phase, Dutch participants categorized a subsequent test continuum as more trochee-like. Moreover, the *selective adaptation* and recalibration effects generalized to novel words, not encountered during exposure. Together, the experiments demonstrate that listeners also flexibly adapt to variability in the suprasegmental properties of speech, thus expanding our understanding of the utility of listener adaptation in speech perception. Moreover, the combined outcomes speak for an architecture of spoken word recognition involving abstract prosodic representations at a prelexical level of analysis.

## 1 Introduction

The speech produced by the talkers we encounter in our everyday lives is highly variable: each talker has its own pronunciation habits. Moreover, the same acoustic cues may signal different speech sounds for different talkers. For instance, one talker’s pronunciation of the vowel /ɪ/ may be acoustically very similar to another talker’s /ɛ/ (Ladefoged & Broadbent, 1957). One perceptual mechanism that helps listeners cope with this variability is *adaptation*: listeners may adjust perceptual boundaries between sound categories in response to previous exposure to a given talker’s speech. The present paper demonstrates that two types of such listener adaptation—*selective adaptation* and *recalibration*—also extend to suprasegmental cues to lexical stress.

*Selective adaptation* is an effect that involves repeated exposure to a stimulus, which induces a perceptual retuning such that the perception of following ambiguous target stimuli is biased *away* from the exposure stimulus. For instance, exposure to a repeatedly presented /ba/ biases perception of a following acoustic /ba-da/ continuum towards /da/ (Eimas & Corbit, 1973). This effect was initially interpreted in terms of fatiguing feature detectors (Eimas & Corbit, 1973), but later studies challenged this view (Remez, 1987; Samuel, 1986). Although the specific kind of retuning involved in *selective adaptation* is debated (Bowers et al., 2016; Kleinschmidt & Jaeger, 2015, 2016; Mitterer et al., 2018; Samuel, 2020), it is generally assumed to operate at multiple different levels, ranging from low-level auditory processing all the way up to decision-making (Remez, 1980; Samuel & Kat, 1996), thus supporting the representation of sensory information (Kleinschmidt & Jaeger, 2016).

The *recalibration* paradigm (otherwise known as perceptual learning or phonetic retuning) also involves exposure stimuli that influence subsequent perception of a target continuum. However, critically, the exposure stimulus is acoustically ambiguous between two sound categories, yet it is disambiguated by context. For instance, if an ambiguous sound “?” midway between /f/ and /s/ is repeatedly heard in an /s/-biasing context (e.g., “platypu?”), the perception of a following /f-s/ test continuum is biased towards /s/. Conversely, hearing the same ambiguous sound in /f/-biasing contexts (e.g., “gira?”) biases the perception of the same test continuum towards /f/ (Norris et al., 2003). Recalibration is typically interpreted in terms of adjustment of category boundaries induced by lexical (Norris et al., 2003), visual (Bertelson et al., 2003), semantic (Jesse, 2021), orthographic (Keetels et al., 2016), and even contra-aural context (Scott, 2020). It involves robust perceptual adjustments since recalibration effects generalize to new words not encountered in exposure (McQueen et al., 2006), are detectable very early in perception suggesting a locus at a prelexical level (Mitterer & Reinisch, 2013), and have a lasting influence even after 12 hours outside the laboratory (Eisner & McQueen, 2006). Thus, recalibration seems to serve a vital function in spoken word recognition, allowing listeners to flexibly apply previously learnt knowledge about a talker’s pronunciation patterns to new encounters (for reviews, see Kleinschmidt & Jaeger, 2015; Samuel & Kraljic, 2009).

Most of the literature on listener adaptation has focused on adaptation to *segmental* idiosyncrasies. However, how talkers produce the *prosody* of spoken language is also highly variable. For instance, produced pause distributions, speech rate, and fundamental frequency (F0) patterns vary as a function of talker, dialect, gender, and register (Clopper & Smiljanic, 2011; Quené, 2008; Xie et al., 2021), although—regrettably—little is known about specifically talker variation in lexical stress production. In spite of this prosodic variability, suprasegmental speech cues play a significant role in the recognition of spoken words, including intonation (Kurumada et al., 2014; Xie et al., 2021), speech rate (Bosker et al., 2020; Maslowski et al., 2019), and lexical stress (Cutler & Donselaar, 2001; Jesse et al., 2017)—the focus of the present study. For instance, acoustic cues to lexical stress may distinguish minimal words pairs, such as the noun *OBject* from the verb *obJECT* (uppercase letters indicate stress) in English, or *CAnon* /ˈka:.nɔn/ “canon” versus *kaNON* /ka:.ˈnɔn/ “cannon” in Dutch (Cutler & Donselaar, 2001). But lexical stress even influences word recognition for words that do not form such rare minimal word pairs. For instance, Reinisch et al. (2010) used eye-tracking to assess Dutch listeners’ processing of suprasegmental cues to lexical stress in an online fashion. When presented with four words on screen, including the segmentally overlapping word pair *OCtopus* and *okTOber*, and spoken instructions to “Click once more on the *OCtopus*,” Dutch participants already preferentially fixated *OCtopus* well before hearing the segmentally disambiguating /p/ in the third syllable. This finding, replicated in English (Jesse et al., 2017) and Italian (Sulpizio & McQueen, 2012), demonstrates that listeners use acoustic cues to lexical stress immediately and incrementally to support and constrain spoken word recognition.

Given the critical role of prosody in spoken word recognition, it is all the more surprising that little is known about how listeners cope with variation in the *suprasegmental* properties of speech, compared to segmental variation. Some have suggested that listeners’ adaptation mechanisms operate not only on segmental variation, but they also apply to larger perceptual units, including prosodic representations (McQueen & Dilley, 2021; Mitterer et al., 2011, 2018; Poellmann et al., 2014; Xie et al., 2021). In fact, some state that “the perceptual system is omnivorous—if the input consistently includes a particular pattern, that pattern can be learned as a “chunk,” and such chunks will be used to recognize speech” (Samuel, 2020, p. 11), suggesting that listener adaptation applies equally to segmental and suprasegmental variability. Still, the perception of prosodic categories is typically based on a multidimensional acoustic space involving a combination of co-varying acoustic features (e.g., F0 height, F0 contours, relative duration, and intensity), typically spanning several syllables or words. Perhaps listener adaptation to such distributed and multidimensional prosodic variation is harder or more taxing for the perceptual system. But then again, some segmental contrasts also involve a high-dimensional acoustic space that is likewise distributed over time (McMurray & Jongman, 2011; Szostak & Pitt, 2013). In any case, evidence for adaptation to distributed prosodic variation would require larger abstract prelexical representations than specified in current abstractionist models (McClelland & Elman, 1986; Norris & McQueen, 2008).

At the same time, if such adaptation to prosodic variation could be established, it would provide a promising tool to study the scope of listener adaptation, specifically with respect to learning generalization. That is, studies on segmental adaptation have observed that listeners generalize learnt knowledge about a segmental idiosyncrasy encountered in word X in an exposure phase (e.g., ambiguous fricative “?” encountered in “platypu?”) to their perception of novel word Y at test (e.g., categorizing “nai?” as *nice*, not *knife*) (McQueen et al., 2006). In a similar vein, the generalization of prosodic adaptation could be tested using segmentally distinct materials in exposure and test, gauging whether exposure to a particular prosodic pattern on word X influences the perception of an entirely differently sounding word Y at test.

The present study investigated whether listeners adapt to variation in the *suprasegmental* properties that cue lexical stress, much like they adjust their phoneme boundaries in order to deal with *segmental* variation. There is already evidence to suggest that listeners adapt to variation in larger units than just single segments. For instance, in Dutch, the unstressed prefix “ver-” in *verlossen* /vɛr.ˈlɔ.sə/ “to redeem” may be reduced to [f] in spontaneous speech, leading to confusion with the verb *flossen* /ˈflɔ.sə/ “to floss.” Poellmann et al. (2014) showed that Dutch listeners, when exposed to a “ver”-reducing talker, could learn to recognize [f] as /vɛr/. Moreover, listeners generalized this type of adaptation to new words that had not been heard in exposure, recognizing novel [f.ˈlis] as *verlies* “loss,” not *vlies* “fleece.”

Moreover, it has been shown that listeners can learn to disregard unconventional prosodic contours for a specific talker (Roettger & Rimland, 2020). Listeners can also learn that a particular non-native talker consistently produces lexical stress on the wrong syllable, generalizing that information to the perception of novel words (Reinisch & Weber, 2012). Two studies specifically targeted adaptation to suprasegmental variation using the recalibation paradigm. Mitterer et al. (2011) presented two groups of Mandarin Chinese speakers with the same ambiguous F0 contour, which was semantically and orthographically disambiguated to signal lexical tone 1 for one group, but lexical tone 2 for another. At test, the first group categorized an acoustic continuum ranging from tone 1 to tone 2 as more tone-1-like, while the second group categorized the same continuum as more tone-2-like. This effect even generalized to novel words with different segmental content that had not been encountered in exposure. Kurumada et al. (2018) tested recalibration of the perception of intonational contours in the construction *It looks like an X*. When produced with a pitch accent on the final noun, this phrase carries an affirmative meaning (“*It looks like a zebra*, and it is one”), but when produced with a pitch accent on the verb, it carries a negative meaning (“*It looks like a zebra*, but actually it isn’t”). One group of listeners (“negative bias” group) was presented with clear “affirmative” contours, but also ambiguous versions of this construction, roughly midway between the “affirmative” and “negative” intonation contours, that were disambiguated by a negative continuation (“. . . but it is not”). A second group (“affirmative bias” group) heard clear “negative” contours, but also ambiguous versions that were disambiguated by an affirmative continuation (“. . . and it is one”). As a result, the participants in the “negative bias” group learned to interpret ambiguous intonation contours as carrying the negative meaning, while the “positive bias” group interpreted the same ambiguous contours as carrying the affirmative meaning. Thus, listeners were shown to adapt to prosodic information in order to efficiently and reliably process variable acoustic cues to speech prosody.

Here we tested listener adaptation to variation in suprasegmental cues to lexical stress, targeting both *selective adaptation* and recalibration. We tested Dutch participants since lexical stress in Dutch is primarily cued using suprasegmental cues (F0, intensity, and duration; Rietveld & Van Heuven, 2009)—unlike in English, where in addition to suprasegmental cues, unstressed vowels are often strongly reduced (Braun et al., 2011; Connell et al., 2018).

Experiment 1 targeted *selective adaptation*: Dutch participants passively listened to 24 exposure stimuli, after which they categorized six test stimuli sampled from a lexical stress continuum of the minimal word pair *CAnon* /ˈka:.nɔn/ “canon” versus *kaNON* /ka:.ˈnɔn/ “cannon.” Exposure stimuli involved either: (a) disyllabic words with a trochaic stress pattern (i.e., a strong–weak prosodic pattern; e.g., *KAper* /ˈka:.pər/ “hijacker”); (b) disyllabic words with an iambic stress pattern (i.e., weak–strong; e.g., *kaPEL* /ka:.ˈpɛl/ “chapel”); or (c) monosyllabic control words (e.g., *kaas* /ka:s/ “cheese”). The current studypredicted that repeatedly hearing strong–weak words in exposure would bias perception of the following test stimuli *away* from the strong–weak prosodic pattern, hence leading to a lower proportion of strong–weak responses at test (i.e., fewer *CAnon* responses). Conversely, hearing weak–strong words in exposure would lead to an increase in the proportion of strong–weak responses at test (i.e., more *CAnon* responses). We also expected that the monosyllabic control condition would fall in between the strong–weak and weak–strong condition, since monosyllabic words are not as informative about how a given talker produces lexical stress as multisyllabic words—despite the fact that the suprasegmental characteristics of monosyllabic *kaas* are similar to those of the stressed syllable *KA-* in *KAper*. Finally, two versions of Experiment 1 were tested. In the “segmental overlap” version, exposure and test words overlapped segmentally (all starting with /ka-/). In the “generalization” version, exposure words did not have any segmental overlap with the critical test word pair (no /ka/-initial words in exposure), thus assessing whether the *selective adaptation* to suprasegmental speech cues would generalize across differential segmental content.

Experiment 2 targeted recalibration: two groups of Dutch participants first passively listened to exposure stimuli, after which they categorized the same test stimuli as in Experiment 1. In the exposure phase, the “weak–strong-bias” group heard an acoustically ambiguous auditory stimulus, midway between *CAnon* and *kaNON*, which was disambiguated by seeing the orthographic word “kanon” on screen. In addition, they heard acoustically clear versions of *CAnon* with “canon” on screen. Conversely, the “strong–weak-bias” group heard the same acoustically ambiguous auditory stimulus in the exposure phase but this time combined with orthographic “canon” on screen, and also acoustically clear versions of *kaNON* with orthographic “kanon” on screen. Thus, the weak–strong bias group was predicted to learn to interpret the ambiguous acoustic exposure stimulus as a weak–strong prosodic pattern, while the strong–weak bias group would learn to interpret the same ambiguous stimulus as a strong–weak prosodic pattern. As a result, the two groups would categorize the test continuum differently: the weak–strong-bias group will categorize the test items as more weak–strong-like (fewer strong–weak responses), while the strong–weak-bias group will categorize the test items as more strong–weak-like (more strong–weak responses).

Moreover, again different versions of the experiment were run. In the “segmental overlap” version, the exposure word pair was the same as the test word pair (*CAnon–kaNON*). In the “generalization” version, a different exposure word pair was used (*SERvisch* /ˈsɛr.vis/ “Serbian” vs. *serVIES* /sɛr.ˈvis/ “tableware”), thus assessing whether recalibration would generalize across different segmental content. Finally, the “non-word control” version assessed whether the difference between the two participant groups was indeed driven by recalibration induced by the ambiguous items in exposure, or by *selective adaptation* induced by the “clear” items in exposure. That is, the strong–weak-bias group in the “segmental overlap” version was, in the exposure phase, presented with ambiguous tokens for *CAnon* but also unambiguous tokens of *kaNON*. Exposure to these unambiguous tokens of *kaNON* could in principle also drive a bias towards strong–weak perception at test through *selective adaptation* (cf. Norris et al., 2003). To disentangle the contribution of *selective adaptation* induced by the “clear” tokens versus recalibration induced by the ambiguous tokens to the observed group differences at test, the “non-word control” version was run. The strong–weak-bias group in this version heard unambiguous tokens of *kaNON* and, critically, suprasegmentally ambiguous tokens of the Dutch non-word *losep* /lo:.sɛp/. Conversely, the weak–strong-bias group in the “non-word control” version heard unambiguous tokens of *CAnon* and the same ambiguous tokens of the Dutch non-word *losep*. Since Dutch participants do not have any lexical knowledge about the stress pattern on the Dutch non-word *losep*, we reasoned that hearing the ambiguous suprasegmental cues to lexical stress on *losep* would not be able to induce recalibration. Hence, it was predicted to find no difference between the two participants’ groups in the “non-word control” version of Experiment 2.

## 2 Experiment 1

Experiment 1 targeted evidence for *selective adaptation* to suprasegmental cues to lexical stress in Dutch. The design was adopted from Mitterer et al. (2018). Participants first passively listened to either all strong–weak words, all weak–strong words, or monosyllabic controls (exposure) after which they categorized stimuli from a lexical stress continuum (test). It was predicted that exposure to strong–weak words would bias perception away from the strong–weak prosodic pattern at test, while weak–strong words would lead to an increase in strong–weak responses at test (relative to control). Moreover, manipulating the segmental overlap between exposure and test words (in the “segmental overlap” vs. “generalization” versions) may reveal potential generalization of suprasegmental *selective adaptation* across distinct segmental content.

### 2.1 Method

#### 2.1.1 Participants

Forty-eight participants were recruited from the Max Planck Institute for Psycholinguistics participant pool. Twenty-four participants, 18 females, 6 males; mean age = 22, range = 19–27, were assigned to the “segmental overlap” version and the other twenty-four to the “generalization” version of the experiment, 15 females, 9 males; mean age = 24, range = 19–36. Participants in all experiments reported in this study gave informed consent as approved by the Ethics Committee of the Social Sciences department of Radboud University (project code: ECSW-2019-019).

#### 2.1.2 Materials and design

For the test stimuli in Experiment 1, one Dutch disyllabic minimal pair whose two members only differed in lexical stress were selected: *canon* /ˈka:.nɔn/ “canon” versus *kanon* /ka:.ˈnɔn/ “cannon.” For the exposure stimuli in the “segmental overlap” version of Experiment 1, various sets of words that all started with the phonemes /ka:/ were selected, thus having segmental overlap with the test minimal pair. 12 disyllabic Dutch words with a trochaic stress pattern (strong–weak; e.g., *KAper* /ˈka:.pər/ “hijacker”) and 12 disyllabic Dutch iambs (weak–strong; e.g., *kaPEL* /ka:.ˈpɛl/ “chapel”) were selected. In addition, 12 monosyllabic Dutch control words were selected (e.g., *kaas* /ka:s/ “cheese”). For the “generalization version” of Experiment 1, 12 strong–weak (e.g., *VIsum* /ˈvi.zʏm/ “visa”), 12 weak–strong (e.g., *boeKET* /bu.ˈkɛt/ “bouquet”), and 12 control words (e.g., *ring* /rɪŋ/ “ring”) that *did not* have any segmental overlap with the test minimal pair were selected. See Tables S1–S2 in the Online Supplementary Information for complete lists of all exposure words in either version of Experiment 1 and Figures S1–S2 in the Online Supplementary Information for their suprasegmental properties (duration, intensity, and F0).

**Figure 1.**
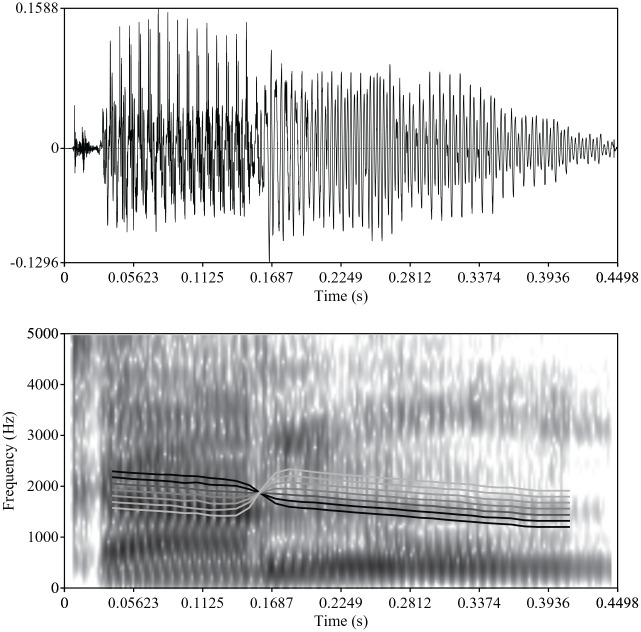
Illustration of the lexical stress continuum from *CAnon* to *kaNON*. An oscillogram and spectrogram are given for the most ambiguous step 4 of the continuum. The lines in the spectrogram indicate the fundamental frequency trajectory from step 1 (black) to step 7 (white) on a scale from 0 to 300 Hz.

A male native speaker of Dutch was recorded with a Sennheiser ME64 directional microphone (audio sampling frequency: 48 kHz) producing all words listed above in isolation. Exposure words were excised from the recordings. For the *CAnon–kaNON* test stimuli, a 7-step lexical stress continuum was created ranging from a “strong–weak” prosodic pattern (step 1) to a “weak–strong” prosodic pattern (step 7), with ambiguous tokens in between. In Dutch, lexical stress is cued by three suprasegmental prosodic cues: F0; duration; and intensity (Rietveld & Van Heuven, 2009). Therefore, an F0 continuum of lexical stress was created while keeping duration and intensity constant at ambiguous values (see Figure 1).

First, the average duration and intensity values were measured separately for the first and second syllables, across the stressed and unstressed versions of the minimal pair: mean duration syllable 1 = 162 milliseconds (ms); syllable 2 = 288 ms; mean intensity syllable 1 = 66.01 dB; and syllable 2 = 66.14 dB. Then, using Praat (Boersma & Weenink, 2021), the strong–weak *CAnon* recording was manipulated by setting the duration and intensity values of the two syllables to these ambiguous values. Subsequently, F0 was manipulated along a 7-step continuum, with the two extremes and the step size informed by the talker’s originally produced F0. Manipulations were always performed in an inverse manner for the two syllables: while the mean F0 of the first syllable decreased along the continuum (from 137 to 113 Hz in steps of 4 Hz), the mean F0 of the second syllable increased (from 108 to 132 Hz in steps of 4 Hz). Moreover, rather than setting the F0 within each syllable to a fixed value, more natural output was created by including a fixed F0 declination within the first syllable (linear decrease of 10 Hz) and second syllable (15 Hz) around the mean value. Pilot data from 12 native Dutch listeners (who did not participate in any of the other experiments) performing a categorization task on these manipulated stimuli showed that the 7-step F0 continuum appropriately sampled the strong–weak to weak–strong perceptual space (see Figure S3 in the Online Supplementary Information): step 1 was relatively strong–weak-like with 0.85 proportion strong–weak responses (*P*(strong–weak)); and step 7 was relatively weak–strong-like with 0.29 *P*(strong–weak). From this continuum the steps 3–5 were selected, which spanned the ambiguous range with *P*(strong–weak) = 0.81, 0.58, and 0.25, respectively.

#### 2.1.3 Procedure

The experimental procedure was modeled after the *selective adaptation* design in Mitterer et al. (2018). Participants were tested individually in a sound-conditioning booth. They were seated at a distance of approximately 60 cm in front of a 50.8 cm × 28.6 cm screen and listened to stimuli at a comfortable volume through headphones. Stimulus presentation was controlled by Presentation software (v16.5; Neurobehavioral Systems, Albany, CA, USA).

Participants were instructed to first passively listen to 24 words and then to identify six additional words by button press (two-alternative forced choice; 2AFC). The experiment was divided in three blocks, one for each exposure condition: strong–weak; weak–strong; or control (order counter-balanced across participants). Each block consisted of 10 cycles, each consisting of the auditory presentation of 24 exposure words, followed by six test words. The exposure words included two repetitions of each of the 12 exposure items for that condition in random order (interstimulus interval = 600 ms; static fixation cross on screen). The test words included two repetitions of each of the three continuum steps in random order. Random orders of exposure words and test words were generated for each cycle anew. The test words were presented with a static fixation cross on screen, which at sound offset was replaced by two response options on either side of the screen: *canon* (with stress on first syllable); and *kanon* (stress on second syllable; position of response options counter-balanced across participants). The participants’ task was to indicate whether the test stimulus had stress on the first or the second syllable (2AFC) by pressing [Z] on a regular keyboard for the left option and [M] for the right option. After their response, or timeout after 3 seconds, the next test stimulus (or the first exposure stimulus of the next cycle after the last test stimulus) was presented after 1350 ms.

Each participant completed 30 cycles in total, each containing 24 exposure and six test stimuli. As a result, 180 test responses were collected from each participant. Participants were given the opportunity to take a short break in between blocks. Note that the two versions of Experiment 1 only differed in the exposure words; the test stimuli were identical in both versions.

### 2.2 Results

**Figure 2.**
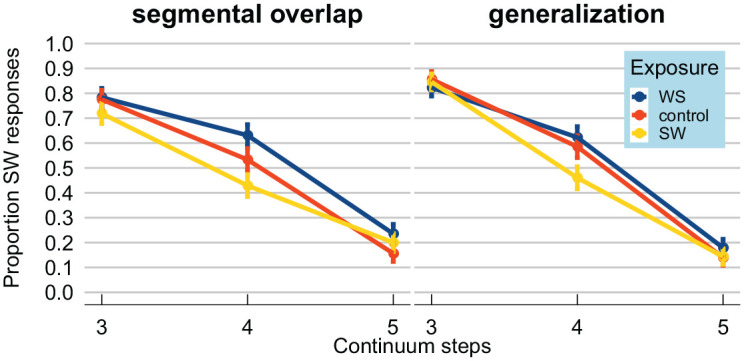
Results from the “segmental overlap” and “generalization” versions of Experiment 1, both targeting selective adaptation. Proportion of test stimuli for which participants reported perceiving lexical stress on the first syllable (i.e., strong–weak; *CAnon*). Test stimuli involved three steps from a lexical stress continuum from *CAnon* (strong–weak) to *kaNON* (weak–strong), varying fundamental frequency independently for the two syllables. Test stimuli were either preceded by exposure words with stress on the first (strong–weak; yellow/light gray) or the second syllable (weak–strong; blue/dark gray), or monosyllabic controls (red/gray). Test stimuli were more likely to be perceived as having stress on the first syllable (strong–weak) if preceded by weak–strong exposure words (compared to strong–weak exposure words): a *selective adaptation* effect. This pattern held both for exposure words with segmental overlap (left panel) and without segmental overlap with the test stimuli (right panel). Error bars enclose 1.96 × standard error on either side; that is, the 95% confidence intervals over the entire dataset.

Categorization data from the test stimuli were visualized by calculating proportions of strong–weak responses, presented in Figure 2. As expected, higher steps on the F0 continuum led to fewer strong–weak responses (lines have a negative slope). In the left panel, showing the results from the “segmental overlap” version of Experiment 1, the difference between the blue/dark gray line (strong–weak exposure words, with lexical stress on the first syllable) and the yellow/light gray line (weak–strong exposure words, with lexical stress on the final syllable) seems to demonstrate a *selective adaptation* effect. That is, test stimuli were perceived as more strong–weak-like when they were preceded by weak–strong exposure words. The control condition (in red/gray) seems to fall in between the weak–strong and strong–weak conditions. These patterns seem similar across both the “segmental overlap” and the “generalization” version (right panel) of Experiment 1.

Data were statistically analyzed using a generalized linear mixed model (GLMM; Quené & Van den Bergh, 2008) with a logistic linking function as implemented in the lme4 library (version 1.0.5; Bates et al., 2015) in R (R Development Core Team, 2012). The binomial dependent variable was participants’ categorization of the test stimulus as either having lexical stress on the first syllable (strong–weak; *CAnon*; coded as 1) or the second syllable (weak–strong; *kaNON*; coded as 0). Fixed effects were Continuum Step (continuous predictor; *z*-scored using the function scale() in R to improve model fitting), Exposure Condition (categorical predictor; with the control condition mapped onto the intercept), and Version (categorical predictor using deviance coding; “segmental overlap” coded as -0.5 and “generalization” coded as +0.5), and all interactions. Larger models with Cycle Number (1–10; mean-centered) or Test Trial Number (1–6; mean-centered) did not improve the model fit to the data as tested by log-likelihood ratio tests, and were therefore not included in the final analysis. For visual inspection of any order effects, please refer to Figures S4–S5 in the Online Supplementary Information. The model also included Participant as a random factor, with by-participant random slopes for Continuum Step and Exposure Condition, as advocated by Barr et al. (2013). A model with their interaction included as a by-participant random slope failed to converge.

The model showed a significant effect of Continuum Step, *β* = -1.837, standard error (*SE*) = 0.179, *z =* -10.264, *p <* 0.001, indicating that higher continuum steps led to lower proportions of strong–weak responses. Note that, considering the present coding of the various predictors, this model estimate should only be interpreted with respect to the control conditions in the experiment. The model also showed significant differences between the various Exposure Conditions. That is, the strong–weak exposure condition led to significantly lower proportions of strong–weak responses, compared to control; *β* = -0.366, *SE* = 0.118, *z =* -3.100, *p =* 0.002, and the weak–strong exposure condition led to significantly higher proportions of strong–weak responses, compared to control; *β* = 0.235, *SE* = 0.091, *z =* 2.593, *p =* 0.010. An interaction between Continuum Step and the contrast between control and strong–weak, *β* = 0.184, *SE* = 0.078, *z =* 2.348, *p =* 0.019, and between Continuum Step and the contrast between control and weak–strong, *β* = 0.231, *SE* = 0.077, *z =* 2.986, *p =* 0.003, was also observed. These interactions suggested that the effect of Continuum Step was less pronounced in the strong–weak and weak–strong exposure conditions. However, no other interactions were observed, suggesting that the effects were not modulated by Version.

### 2.3 Interim summary

The results from Experiment 1 demonstrated evidence for *selective adaptation* to suprasegmental cues to lexical stress in Dutch. That is, repeatedly hearing disyllabic Dutch words with a strong–weak stress pattern biased perception of a following suprasegmental lexical stress continuum *away* from the exposure stress pattern relative to control. The opposite held for exposure to Dutch words with a weak–strong prosodic pattern. Moreover, qualitatively similar *selective adaptation* was established for the two versions of Experiment 1, suggesting that *selective adaptation* to suprasegmental speech cues generalizes across differential segmental content.

## 3 Experiment 2

Experiment 2 targeted evidence for recalibration of the perception of suprasegmental cues to lexical stress in Dutch. The experiment was modeled after Keetels et al. (2016) but using a between-participant design as in Norris et al. (2003). Two groups of participants passively listened to 48 exposure words with orthographic word forms on screen after which they received 45 test trials, involving categorization of the same lexical stress continuum as in Experiment 1. The strong–weak-bias group was presented with ambiguous exposure items that were disambiguated by the orthographic word form on screen to indicate a strong–weak pattern (e.g., “canon”). The weak–strong-bias group heard the same ambiguous exposure items but this time disambiguated by the orthographic word form on screen to indicate a weak–strong pattern (e.g., “kanon”). It was predicted that this difference in exposure would bias the strong–weak-bias group towards strong–weak responses at test, while the weak–strong-bias group would show reduced strong–weak responses at test. Again, varying the segmental overlap between exposure and test words (in the “segmental overlap” vs. “generalization” versions) may reveal potential generalization of suprasegmental recalibration across distinct segmental content. Finally, to demonstrate that the group effect is indeed driven by recalibration, the “non-word control” version included ambiguous suprasegmental cues to stress on a Dutch non-word. Since Dutch participants do not have any lexical knowledge about the stress patterns on non-words, no recalibration is predicted in this control version of Experiment 2.

### 3.1 Method

#### 3.1.1 Participants

One hundred and three new participants were recruited from the Max Planck Institute for Psycholinguistics participant pool. Thirty-six of these were assigned to the “segmental overlap” version, 31 females, 5 males; mean age = 23, range = 18–29, thirty-five to the “generalization” version, 26 females, 9 males; mean age = 24, range = 18–35, and thirty-two to the “non-word control” version, 29 females, 3 males; mean age = 22, range = 18–27.

#### 3.1.2 Materials and design

For the test stimuli used in all versions of Experiment 2, the same three steps were used as the ones used in the test phase in Experiment 1, namely steps 3–5. For the exposure stimuli of the “segmental overlap” version of Experiment 2, three manipulated tokens from the original 7-step F0 continuum from *CAnon* (strong–weak) to *kaNON* (weak–strong) were selected, created for Experiment 1 (cf. Figure S3 in the Online Supplementary Information). Step 1 was selected as “clear” strong–weak item, step 4 was selected as ambiguous item, and step 7 was selected as “clear” weak–strong item.

For the “generalization” and “non-word control” versions of Experiment 2, different exposure stimuli were used. For the “generalization” version, the same male native speaker of Dutch was recorded, using the same audio equipment, producing the minimal lexical stress pair *SERvisch* /ˈsɛr.vis/ “Serbian” (with stress on the first syllable) versus *serVIES* /sɛr.ˈvis/ “tableware” (with stress on the second syllable) in isolation. For the “non-word control” version, the non-word minimal pair *LOsep* /ˈlo:.sɛp/ versus *loSEP* /lo:.ˈsɛp/ was recorded from the same speaker.

From these new recordings, 7-step F0 lexical stress continua were created from the original strong–weak recordings (i.e., *SERvisch* and *LOsep*). The intensity and F0 values were identical to the continuum values from the *CAnon–kaNON* continuum used previously, since these values were close to the mean values across the stressed and unstressed versions of these items. However, because the segmental content and syllabic complexity varied across minimal pairs (i.e., CVC.CVC vs. CV.CVC), duration values were not adopted from the earlier continuum. Instead, the durations of the first and second syllables were set to the average duration calculated across the stressed and unstressed versions, *servisch*: mean duration syllable 1 = 277 ms; syllable 2 = 398 ms; *losep*: syllable 1 = 202 ms; syllable 2 = 395 ms. Pilot data from native Dutch listeners (who did not participate in any of the other experiments) who performed a categorization task on these manipulated stimuli showed that these 7-step F0 continua were perceptually comparable to the *CAnon–kaNON* continuum used previously (see Figure S3 in the Online Supplementary Information). For *servisch*, step 1 was relatively strong–weak-like with 0.85 *P*(strong–weak), step 4 with 0.50 *P*(strong–weak), and step 7 was relatively weak–strong-like with 0.21 *P*(strong–weak). Similarly, for *losep*, step 1 was categorized as 0.85 *P*(strong–weak), step 4 as 0.55 *P*(strong–weak), and step 7 as 0.20 *P*(strong–weak).

#### 3.1.3 Procedure

The experimental procedure was modeled after the recalibration design in Keetels et al. (2016). Experiment 2 was run online using PsyToolkit (version 2.6.1; Stoet, 2017) because of limitations due to the ongoing COVID-19 pandemic. Each participant was explicitly instructed to use headphones and to run the experiment with undivided attention in quiet surroundings.

The experiment was divided into an exposure phase and a test phase. Within each of the three versions, participants were randomly allocated to one of two groups: the weak–strong-bias group; or the strong–weak-bias group. The two groups received different exposure stimuli, but the same test stimuli. In the “segmental overlap” version, the weak–strong-bias group was instructed to passively listen to 48 words whose labels were presented in orthographic form on screen. For this group, exposure items involved 24 repetitions of step 1 (i.e., most strong–weak-like) with the word “canon” on screen and 24 repetitions of the ambiguous step 4 with the word “kanon” on screen. This design prompted participants in the weak–strong-bias group to learn that the talker used the ambiguous suprasegmental cues in step 4 to cue a weak–strong prosodic pattern. Conversely, the strong–weak-bias group was presented with 24 repetitions of step 7 (i.e., most weak–strong-like) with the word “kanon” on screen and 24 repetitions of the ambiguous step 4 with the word “canon” on screen. This design prompted the strong–weak-bias group to learn that the ambiguous suprasegmental cues in step 4 indicated a strong–weak prosodic pattern.

In the “generalization” version, a similar group design was adopted but this time involving steps 1, 4, and 7 from the *SERvisch–serVIES* continuum. That is, once again participants were randomly allocated to either the weak–strong-bias group or the strong–weak-bias group. The weak–strong-bias group was presented with step 1 with the word “Servisch” on screen and step 4 with the word “servies” on screen. By contrast, the strong–weak-bias group was presented with step 7 with the word “servies” on screen and step 4 with the word “Servisch” on screen.

Finally, the exposure phase in the “non-word control” version was similar to the “segmental overlap” version, except that ambiguous suprasegmental cues were only ever presented on the non-word *losep* (i.e., step 4). Specifically, the weak–strong-bias group in the “non-word control” version was presented with 24 repetitions of step 1 from the *kanon* continuum (i.e., most strong–weak-like) with the word “canon” on screen and 24 repetitions of the ambiguous step 4 from the *losep* continuum with the non-word “losep” on screen. Conversely, the strong–weak-bias group was presented with 24 repetitions of step 7 from the *kanon* continuum (i.e., most weak–strong-like) with the word “kanon” on screen and 24 repetitions of the ambiguous step 4 from the *losep* continuum with the non-word “losep” on screen. Thus, the two groups in the “non-word control” version received the same unambiguous tokens as the “segmental overlap” version, but different suprasegmentally ambiguous tokens.

For all versions it held that each exposure stimulus started with a fixation cross on screen for 500 ms, followed by the orthographic stimulus on screen, followed by the auditory stimulus after a 450 ms delay (following Keetels et al., 2016). Participants pressed the ENTER key to move to the next exposure item after sound offset. Exposure stimuli were presented in random order.

The exposure phase was followed by a test phase, involving a 2AFC identification task using the same lexical stress continuum as used in Experiment 1. This test phase was identical in each version of Experiment 2. In total, 15 repetitions of each of the three steps were presented in random order, leading to a total of 45 test trials per participant. Test stimuli were presented with a static fixation cross on screen, which at sound offset was replaced by two response options on either side of the screen: *canon* (with stress on first syllable); and *kanon* (stress on second syllable; position of response options counter-balanced across participants). The participants’ task was to indicate whether the test stimulus had stress on the first or the second syllable by pressing [Z] on a regular keyboard for the left option and [M] for the right option. After their response, or timeout after 3 seconds, the next test stimulus was presented after 1000 ms.

### 3.2 Results

**Figure 3.**
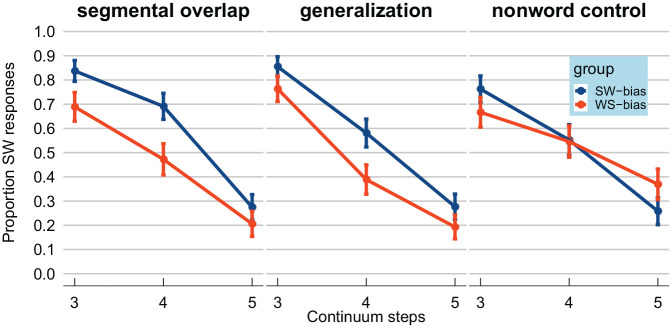
Results from the “segmental overlap,” “generalization,” and “non-word control” versions of Experiment 2, targeting recalibration. Proportion of test stimuli for which participants reported perceiving lexical stress on the first syllable (i.e., strong–weak; *CAnon*). Test stimuli involved three steps from a lexical stress continuum from *CAnon* (strong–weak) to *kaNON* (weak–strong) varying fundamental frequency independently for the two syllables. The strong–weak-bias group (in blue/dark gray) was exposed to suprasegmentally ambiguous strong–weak words, thus learning that ambiguous suprasegmental cues to lexical stress indicate a strong–weak prosodic pattern. Conversely, the weak–strong-bias group (in red/gray) was exposed to suprasegmentally ambiguous weak–strong words, thus learning that ambiguous suprasegmental cues to lexical stress indicate a weak–strong prosodic pattern. In the “segmental overlap” version of Experiment 2, the exposure stimuli shared the same segmental content as the test stimuli (left panel). In the “generalization” version of Experiment 2 (middle panel), the exposure stimuli were taken from a lexical stress continuum from a different minimal pair (*SERvisch* (strong–weak)–*serVIES* (weak–strong)). Across both these versions, the strong–weak-bias group showed more strong–weak responses for the same test stimuli compared to the weak–strong-bias group. Finally, in the “non-word control” version, participants heard the same unambiguous tokens in exposure as the “segmental overlap” version, but only ever heard ambiguous suprasegmental cues to lexical stress on the non-word *losep*—thus preventing recalibration. Error bars enclose 1.96 × standard error on either side; that is, the 95% confidence intervals over the entire dataset.

Missing responses due to time-out were excluded from analysis (*n =* 54; 1%). Categorization data from the test stimuli were visualized by calculating proportions of strong–weak responses, presented in Figure 3. As expected, higher steps on the F0 continuum led to fewer strong–weak responses (lines have a negative slope). In the left panel, results from the “segmental overlap” version of Experiment 2 are presented. The difference between the blue/dark gray line (the strong–weak-bias group) and the red/light gray line (weak–strong-bias group) seems to demonstrate a recalibration effect. That is, the same test stimuli were perceived as more strong–weak-like by the strong–weak-bias group but as more weak–strong-like by the weak–strong-bias group. The patterns in the middle panel, showing the data from the “generalization” version of Experiment 2, look very similar to the left panel, despite the fact that the exposure phase in the “generalization” version involved a segmentally distinct minimal pair. Finally, the right panel shows the data from the “non-word control” version. Here the two lines representing the two groups largely overlap.

Data were statistically analyzed by another GLMM with a logistic linking function. The binomial dependent variable was participants’ categorization of the test stimulus as either having lexical stress on the first syllable (strong–weak; *CAnon*; coded as 1) or the second syllable (weak–strong; *kaNON*; coded as 0). Fixed effects were Continuum Step (continuous predictor; *z*-scored to improve model fitting), Group (categorical predictor using deviance coding; strong–weak-bias group coded as +0.5, weak–strong-bias group coded as -0.5), and Version (categorical predictor; with “segmental overlap” mapped onto the intercept), and all interactions. A larger model with Test Trial Number (1–45; mean-centered) did not improve the model fit to the data as tested by a log-likelihood ratio test and was therefore not included in the final analysis. For visual inspection of any order effects, please refer to Figure S6 in the Online Supplementary Information. The model also included Participant as a random factor, with by-participant random slopes for Continuum Step and Group, as advocated by Barr et al. (2013).

We observed a significant effect of Continuum Step, *β* = -1.296, *SE* = 0.165, *z =* -7.840, *p <* 0.001, indicating that higher continuum steps led to lower proportions of strong–weak responses in the “segmental overlap” version. We also found an effect of Group, *β* = 0.906, *SE* = 0.268, *z =* 3.378, *p <* 0.001, indicating that the strong–weak-bias group demonstrated a significantly higher proportion of strong–weak responses compared to the weak–strong-bias group in the “segmental overlap” version. Critically, no interaction was observed between Group and the “generalization” version, *β* = -0.122, *SE* = 0.376, *z =* -0.326, *p =* 0.745, suggesting a statistically comparable Group difference in the “generalization” version as in the “segmental overlap” version. However, a significant interaction between Group and the “non-word control” version, *β* = -0.907, *SE* = 0.383, *z =* -2.369, *p =* 0.018, demonstrated a significantly reduced Group effect in the “non-word control” version. In fact, judging from the *β* estimates, the Group difference in the “non-word control” version was estimated to be trivial (i.e., 0.906–0.907).

### 3.3 Interim summary

The results from Experiment 2 demonstrated evidence for recalibration of the perception of suprasegmental cues to stress in Dutch. The strong–weak-bias group consistently demonstrated a higher proportion of strong–weak responses at test compared to the weak–strong-bias group. Moreover, qualitatively similar recalibration results were found for the “segmental overlap” and “generalization” versions of Experiment 2, suggesting generalization of suprasegmental recalibration across differential segmental content. Finally, the absence of a group effect in the “non-word control” version confirmed that the group effects in the other two versions were driven by lexical recalibration.

## 4 General discussion

This study demonstrated that listeners adapt to variability in the suprasegmental speech cues that signal lexical stress in Dutch. Dutch was selected as the target language since lexical stress is principally cued by suprasegmental cues (F0, intensity, and duration; Rietveld & Van Heuven, 2009), unlike English where segmental reduction is a primary cue to stress. Experiment 1 showed evidence for a *selective adaptation* effect in lexical stress perception: repeatedly hearing disyllabic Dutch words with a strong–weak stress pattern (i.e., trochees) biased perception of a following suprasegmental lexical stress continuum *away* from the exposure stress pattern; that is, the proportion of strong–weak responses decreased. Conversely, repeatedly hearing disyllabic Dutch words with weak–strong stress patterns (weak–strong; i.e., iambs) increased the proportion of strong–weak responses.

Interestingly, performance on the control condition, involving monosyllabic exposure stimuli, fell roughly in between the strong–weak and weak–strong conditions. This is particularly striking, considering that, in the “segmental overlap” version of Experiment 1, the suprasegmental properties of the /ka/ interval in the monosyllabic control words was very similar to those of the /ka/ interval in the strong–weak condition (cf. Figures S1–S2 in the Online Supplementary Information). Accordingly, one could have predicted that the monosyllabic condition would actually pattern together with the strong–weak condition. However, this was not observed. Instead, the present finding suggests that participants did not merely adapt to the acoustic properties of the exposure words, but actually adapted to the suprasegmental cues that function as signals to lexical stress. That is, even though the suprasegmental cues on the monosyllabic control words mimicked those on the first syllables of the strong–weak words, they were not taken as informative about how the talker produces lexical stress, and hence did not influence perception of the subsequent disyllabic test continuum.

Experiment 2 provided evidence for a *recalibration* effect in lexical stress perception. In the “segmental overlap” version of Experiment 2, two groups passively listened to exposure stimuli, followed by a categorization task on the same suprasegmental test continuum used in Experiment 1. The “weak–strong bias” group heard a suprasegmentally unambiguous strong–weak word (*CAnon*) in the exposure phase, with accompanying orthographic label on the screen (“canon”), as well as a suprasegmentally ambiguous stimulus midway between strong–weak and weak–strong, which was disambiguated by a weak–strong label on screen (“kanon”). The “strong–weak bias” group heard, in their exposure phase, a clear weak–strong word (*kaNON*) with “kanon” on screen, and the same suprasegmentally ambiguous stimulus, but this time disambiguated by a strong–weak label on screen (“canon”). Hence, the “weak–strong bias” group learned to interpret the ambiguous suprasegmental cues as signaling a weak–strong prosodic pattern, while the “strong–weak bias” group learned to interpret the same suprasegmental cues as signaling a strong–weak prosodic pattern. Consequently, the “weak–strong bias” group demonstrated a lower proportion of strong–weak responses on the following suprasegmental test continuum compared to the “strong–weak bias” group.

Outcomes of the “non-word control” version of Experiment 2 revealed that the group effect observed in the “segmental overlap” version could not be attributed to *selective adaptation* to the unambiguous tokens in exposure. That is, participants in the “non-word control” version heard the same unambiguous tokens in exposure as the participants in the “segmental overlap” version. The two versions only differed in whether the ambiguous suprasegmental cues to lexical stress were presented on real words (in the “segmental overlap” version, thus inducing recalibration) or on a non-word (in the “non-word control” version, thus preventing recalibration). The “non-word control” version demonstrated no difference between the two participant groups. Therefore, the group effect in the “segmental overlap” version may be interpreted as primarily indicating evidence for lexically-driven recalibration, induced by exposure to ambiguous suprasegmental cues to lexical stress on real words. Thus, Experiment 1 and 2 together exhibit two different forms—*selective adaptation* and recalibration—of flexible and robust listener adaptation to variability in suprasegmental speech cues to lexical stress.

The underlying cognitive machinery responsible for the recalibration observed in Experiment 2 may involve both interactive and/or feedforward mechanisms. In an interactive framework of spoken word recognition (McClelland & Elman, 1986), the visual orthographic context in exposure would influence the prelexical processing of the ambiguous suprasegmental speech cues in a top-down fashion, thus retuning subsequent prelexical processing at test (Mirman et al., 2006). A feedforward account (Norris & McQueen, 2008) would argue that the visual orthographic context and prelexical representations of lexical stress are combined at the decision level, retuning abstract prelexical representations over time (Norris et al., 2016). Although the present design does not discriminate between these two accounts, the current outcomes do emphasize that any formal account of spoken word recognition should consider listener adaptation not only to variability in the segmental but also the *suprasegmental* content of spoken language.

Importantly, the “generalization” version of Experiment 2 provided evidence for generalization of listener adaptation to suprasegmental variation to new words. That is, listeners used their knowledge about suprasegmental cues to lexical stress acquired in exposure to perceive new words with a different segmental composition at test. This may be compared to generalization of learning about an ambiguous fricative “?”, midway between /s/ and /f/, encountered in “platypu?” in exposure and then applying that knowledge when categorizing novel “nai?” as *nice* (vs. *knife*) at test (McQueen et al., 2006). Generalization of learning to previously unheard words has been argued to indicate that an abstraction process concerning the ambiguous sound in exposure must have taken place at a prelexical level (Cutler et al., 2010; Mitterer et al., 2011). That is, the perceptual adjustments induced by the exposure phase affected a prelexical stage of processing, because it allowed learning to transfer to other words produced by that talker. Thus, these prelexical abstractions help the listener in solving the “lack of invariance” in the speech signal: due to the learning in the exposure phase, listeners know how to interpret the otherwise ambiguous suprasegmental cues in the test phase. As such, the present findings suggest that the representation of prosodic structures, such as lexical stress, is based on phonological abstraction (Honbolygó & Csépe, 2013; Sulpizio & McQueen, 2012), in line with earlier evidence for abstract phonological knowledge about prosody, such as relative syllable durations (Shatzman & McQueen, 2006) and lexical tone (Mitterer et al., 2011). That is, detailed storage of acoustic exemplars alone (as episodic accounts of spoken word recognition would argue; Bybee, 2001; Goldinger, 1998; Pierrehumbert, 2002) is insufficient to account for the present findings.

One possible implementation of how the suprasegmental cues in the acoustic signal are computed at a prelexical level involves a “Prosody Analyzer” (Cho et al., 2007). This analyzer, building an abstract prosodic representation of the spoken input, works in parallel with other prelexical mechanisms responsible for the extraction of segmental cues. Together, these mechanisms constrain lexical access (McQueen & Dilley, 2021). Because in this proposal both segmental and suprasegmental mechanisms are interconnected, the present study demonstrates evidence for listener adaptation to suprasegmental representations—much like what has been found for segments (Kraljic et al., 2008; Norris et al., 2003; Poellmann et al., 2014). An interesting avenue for further research is the comparison of adaptation at the segmental versus suprasegmental level. For instance, a potential candidate brain area identified to be involved in segmental adaptation is the superior temporal sulcus (STS). The STS seems to be instrumental in segmental recalibration induced by visual articulatory context (Kilian-Hütten et al., 2011) and orthographic context (Bonte et al., 2017). Future neuroimaging work may reveal whether the neurobiological machinery involved in segmental and suprasegmental adaptation is shared. Indeed, if, as suggested by Cho et al. (2007), the segmental and suprasegmental analyzers are interconnected, one could predict that similar constraints would hold for adaptation to segmental and suprasegmental variability. Comparing the perceptual locus (Mitterer & Reinisch, 2013), and the stability over time (Eisner & McQueen, 2006) of suprasegmental adaptation to segmental adaptation could provide valuable insight into the relationship between segmental and suprasegmental abstraction.

Moreover, how exactly the listeners in this study adjusted the abstract prelexical representations of lexical stress remains to be examined. For instance, did the “strong–weak-bias” group in Experiment 2 learn that the talker at hand only produced “odd-sounding” suprasegmental cues to specifically disyllabic trochees, or to all words with initial stress? If the latter, one could predict that the “strong–weak-bias” group might generalize their learning in exposure even to trisyllabic words with initial versus penultimate stress (e.g., adjective “foregoing” vs. verb “forgoing” in English). Also, the talker-specific nature of prosodic adaptation is of interest (Xie et al., 2021). For instance, did the listeners in this study’s experiments adjust the perceptual boundary between strong–weak and weak–strong prosodic patterns only for the particular talker at hand (e.g., Eisner & McQueen, 2005), or did they adjust the boundary more generally (Kraljic & Samuel, 2007; Reinisch & Holt, 2014)? Furthermore, what is the role of language-specific biases in prosodic adaptation, for instance comparing languages with different distributional properties of various stress patterns (e.g., Italian vs. Dutch; Sulpizio & McQueen, 2012)? Future studies may assess the precise cognitive adjustments that listeners may make to abstract representations of lexical stress.

Finally, it should be pointed out that this study’s design of the exposure phase in Experiment 2 involved disambiguation of the ambiguous suprasegmental cues to lexical stress by *orthographic labels of lexical items*. Hence, it cannot be distinguished whether the recalibration observed here was driven by lexical recalibration (Norris et al., 2003), orthographic recalibration (FKeete et al., 2016), or (perhaps most likely) a combination of the two. Further experiments may target the individual contribution of lexical and orthographic context to listener adaptation to suprasegmental variability; and of course also other types of context, including visual context. Indeed, recent frameworks of face-to-face spoken communication emphasize the multimodal context in which spoken word recognition takes place (e.g., Holler & Levinson, 2019), including articulatory cues on the face (e.g., wider and longer lip aperture for stressed vs. unstressed syllables; Jesse & McQueen, 2014) as well as co-speech gestures that are typically aligned to the acoustically prominent syllables in speech. Recently, it was shown that listeners exploit the tight temporal relationship between beat gestures and lexical stress in speech production to support speech perception. That is, seeing a beat gesture aligned to a particular spoken syllable biases listeners to perceive that syllable as stressed: a “manual McGurk effect” (Bosker & Peeters, 2021). Following from this finding, demonstrating that simple manual movements aligned to speech prosody are capable of recalibrating abstract representations of LS would be powerful evidence in favor of multimodal views on human communication.

## Supplemental Material

sj-pdf-1-las-10.1177_00238309211030307 – Supplemental material for Evidence For Selective Adaptation and Recalibration in the Perception of Lexical Stress Click here for additional data file. Supplemental material, sj-pdf-1-las-10.1177_00238309211030307 for Evidence For Selective Adaptation and Recalibration in the Perception of Lexical Stress by Hans Rutger Bosker in Language and Speech
