# Supplementary material for: Evidence For Selective Adaptation and Recalibration in the Perception of Lexical Stress
Source: Lang Speech. 2021 Jul 6;65(2):472–90. doi: 10.1177/00238309211030307 (PMC9014674; doi:10.1177/00238309211030307)
Supplement: sj-pdf-1-las-10.1177_00238309211030307 – Supplemental material for Evidence For Selective Adaptation and Recalibration in the Perception of Lexical Stress [file sj-pdf-1-las-10.1177_00238309211030307.pdf]

Supplementary Information

Evidence for selective adaptation and recalibration in the perception of lexical stress

Hans Rutger Bosker

Corresponding author: Hans Rutger Bosker
Email: HansRutger.Bosker@mpi.nl

This file includes:

Supplementary figures
Figure S1-6
Supplementary tables
Table S1-2

Note that the experimental data of this study are publicly available for download from https://osf.io/dfjyn under a CC BY-NC-ND 4.0 license.

Supplementary figures

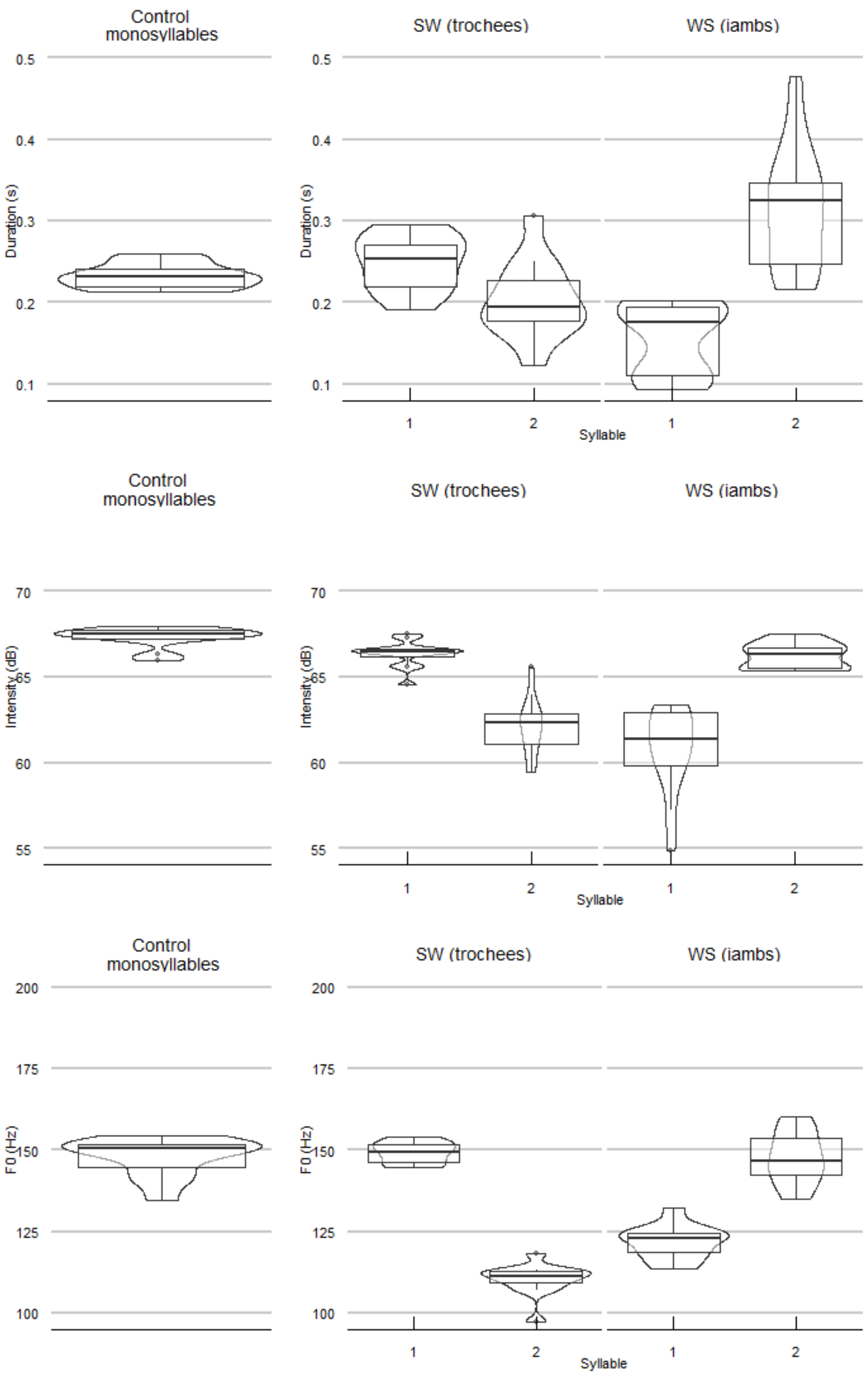

Figure S1. Suprasegmental properties for the exposure stimuli used in the 'segmental overlap' version of Experiment 1. Duration (in ms; top row), overall intensity (in dB; middle row), and F0 values (in Hz; bottom row) for the monosyllabic control words, and the SW and WS disyllabic words, split for each syllable. Note that the measurements of the control monosyllabic words were made on the initial /ka/ segments, excluding the final coda consonant(s), to aid comparison to the first /ka/ syllable of the disyllabic SW and WS words.

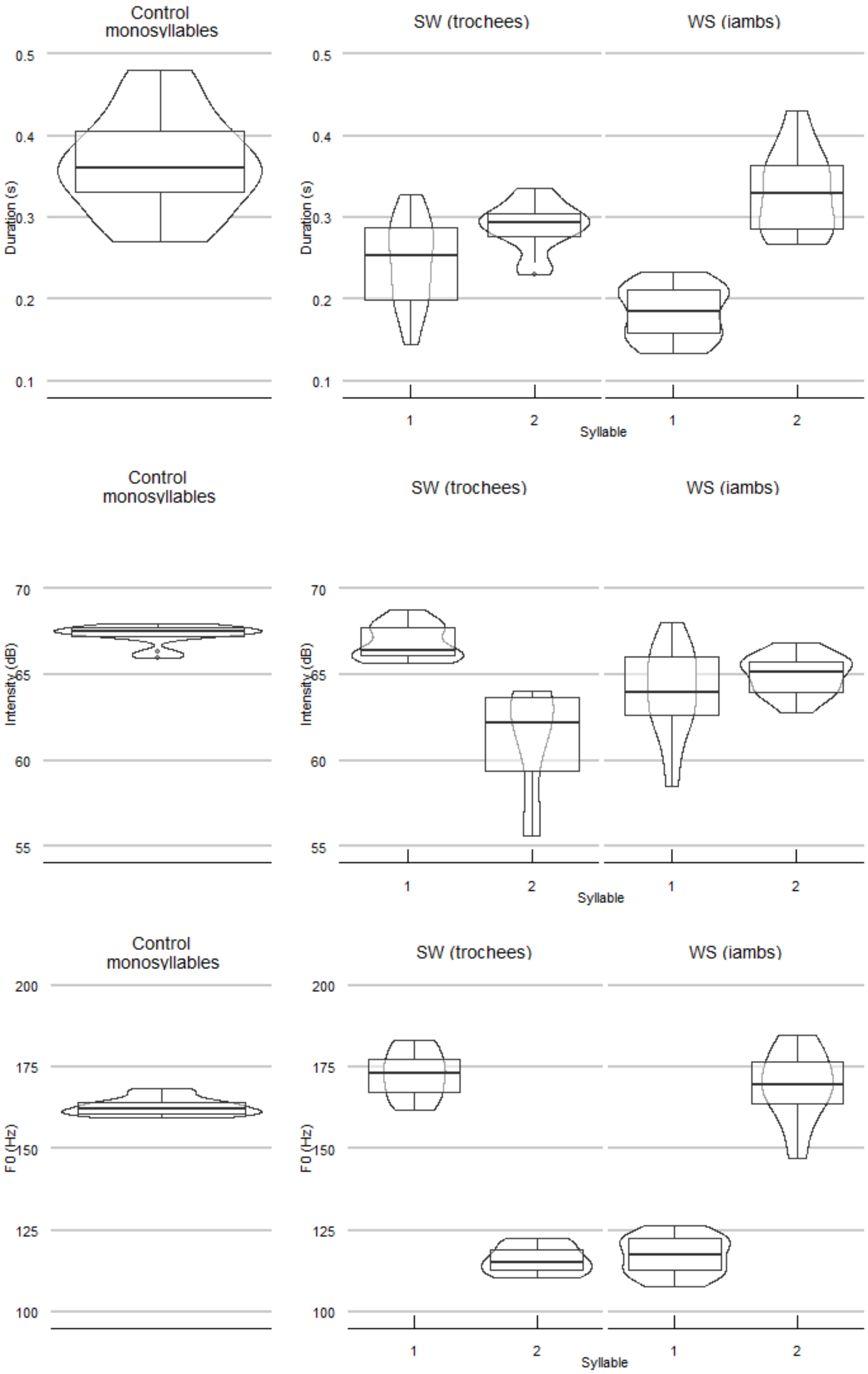

Figure S2. Suprasegmental properties for the exposure stimuli used in the 'generalization' version of Experiment 1. Duration (in ms; top row), overall intensity (in dB; middle row), and F0 values (in Hz; bottom row) for the monosyllabic control words, and the SW and WS disyllabic words, split for each syllable. Note that the measurements of the control monosyllabic words were made on the entire words, also including coda consonants (contrary to the measurements in Figure S1).

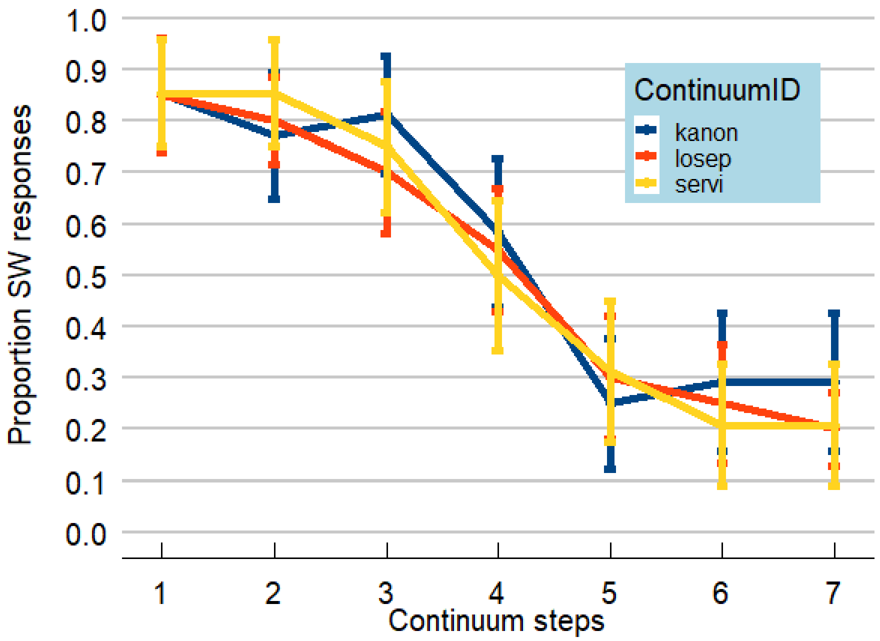

Figure S3. Pilot data for the three lexical stress continua used in Experiment 2. Proportion of trials for which participants reported perceiving lexical stress on the first syllable (i.e., strong-weak) for the *CAnon-kaNON* word pair used in Experiments 1-2 (in blue), for the *LOsep-loSEP* non-word pair used in Experiment 2 ('non-word control' version; in red), and for the *SERvisch-serVIES* word pair used in Experiment 2 ('generalization' version; in yellow). The lines largely overlap indicating that the different phonetic continua are comparable in lexical stress perception. Error bars enclose 1.96 x SE on either side; that is, the 95% confidence intervals over the entire dataset.

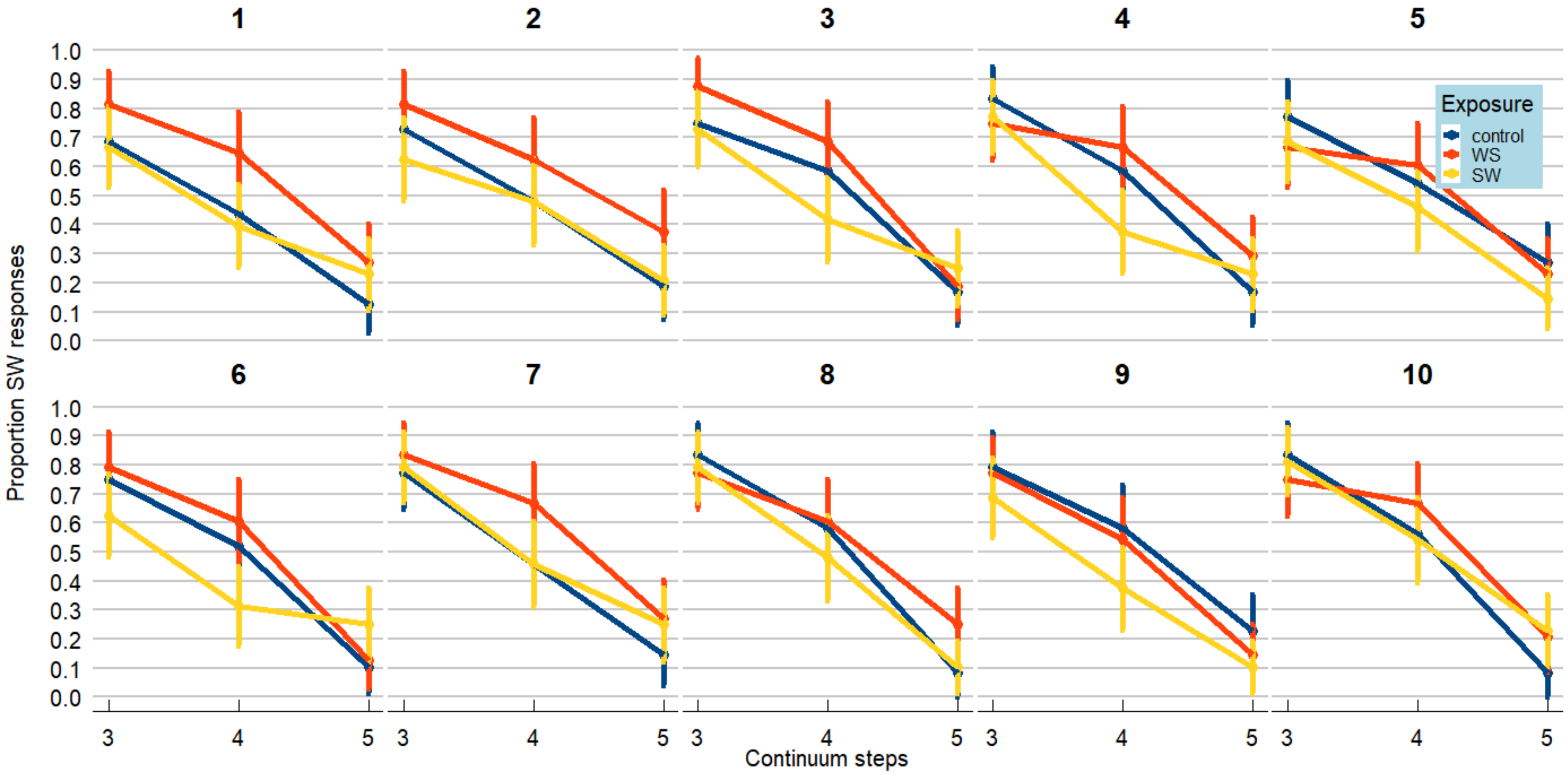

Figure S4. Data from the 'segmental overlap' version of Experiment 1, split by cycle number. Proportion of test stimuli for which participants reported perceiving lexical stress on the first syllable (i.e., strong-weak; *CAnon*). Test stimuli involved 3 steps from a lexical stress continuum from *CAnon* (strong-weak) to *kaNON* (weak-strong), varying F0 independently for the two syllables. Test stimuli were either preceded by exposure words with stress on the first (strong-weak; yellow/lightgray) or the second syllable (weak-strong; blue/darkgray), or monosyllabic controls (red/gray). Participants were presented with 10 cycles of stimuli (digits above each panel), each comprising 24 exposure trials and 6 test trials. Error bars enclose 1.96 x SE on either side; that is, the 95% confidence intervals over the entire dataset.

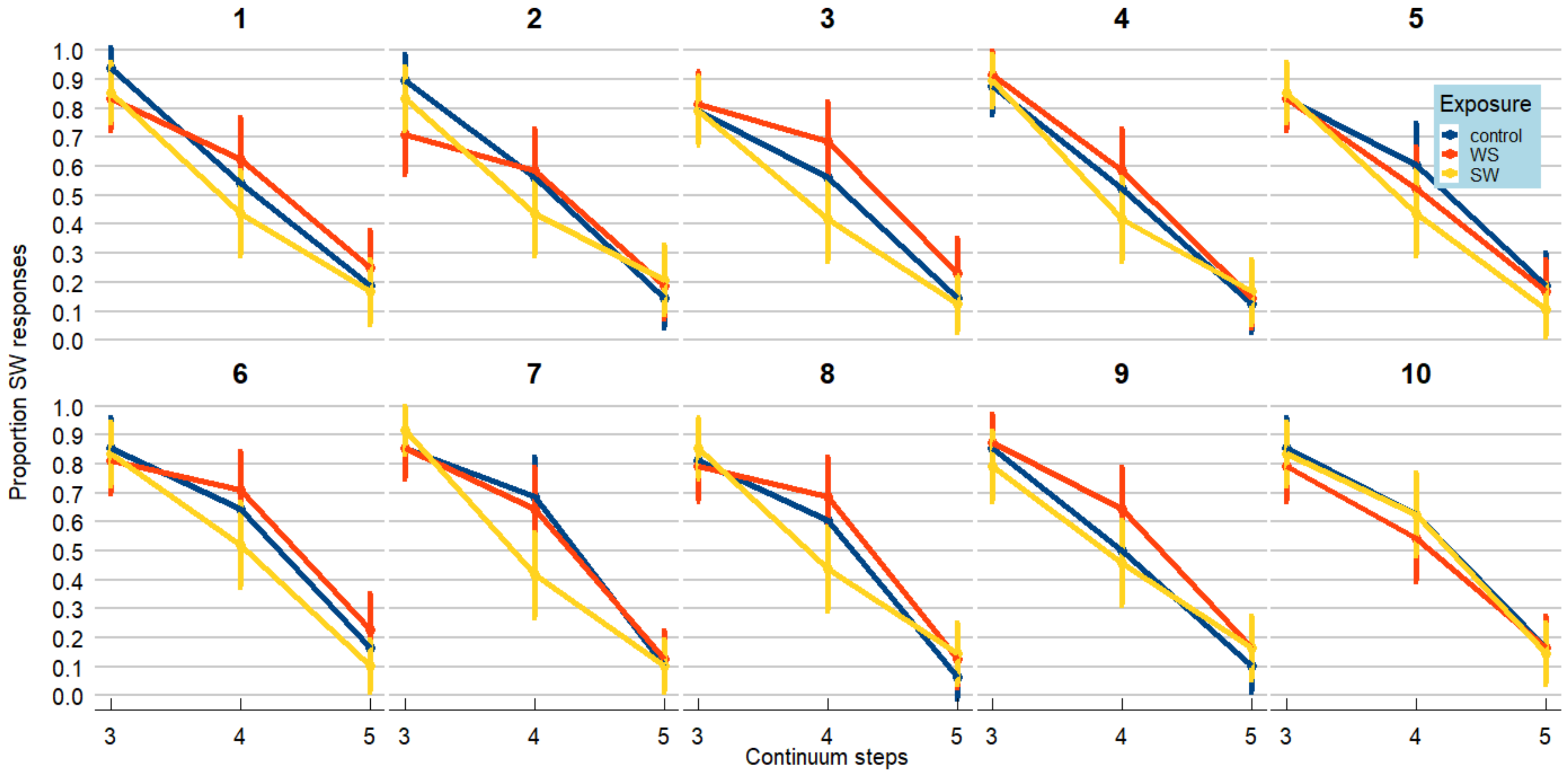

Figure S5. Data from the 'generalization' version of Experiment 1, split by cycle number. Proportion of test stimuli for which participants reported perceiving lexical stress on the first syllable (i.e., strong-weak; *CAnon*). Test stimuli involved 3 steps from a lexical stress continuum from *CAnon* (strong-weak) to *kaNON* (weak-strong), varying F0 independently for the two syllables. Test stimuli were either preceded by exposure words with stress on the first (strong-weak; yellow/lightgray) or the second syllable (weak-strong; blue/darkgray), or monosyllabic controls (red/gray). Participants were presented with 10 cycles of stimuli (digits above each panel), each comprising 24 exposure trials and 6 test trials. Error bars enclose 1.96 x SE on either side; that is, the 95% confidence intervals over the entire dataset.

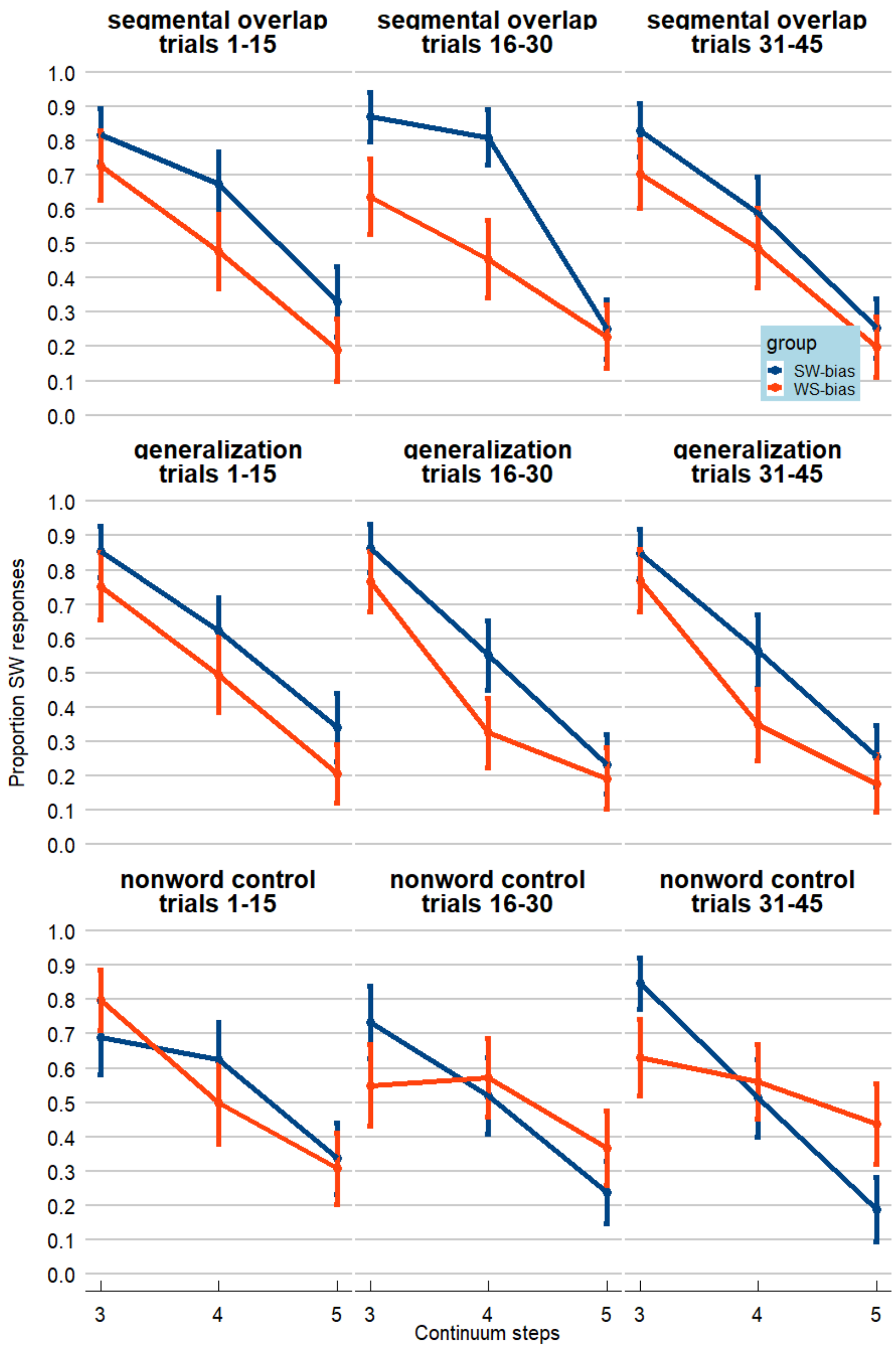

Figure S6. Results from the three versions of Experiment 2, split by the first 15, second 15, and third 15 test trials. Proportion of test stimuli for which participants reported perceiving lexical stress on the first syllable (i.e., strong-weak; CAnon). While there seems to be a consistent group effect in the 'segmental overlap' and 'generalization' versions, there seems to be a consistent null effect in the 'non-word control' version. Error bars enclose 1.96 x SE on either side; that is, the 95% confidence intervals over the entire dataset.

Supplementary tables

Table S1. List of exposure words (in Dutch /IPA/ "English") used in the 'segmental overlap' version of Experiment 1. SW = strong-weak; WS = weak-strong.

Experiment 1A

	**SW**	**WS**	**control**
1	kaper /ˈka:.pər/ "hijacker"	kapel /ka.ˈpɛl/ "chapel"	kaas /ka:s/ "cheese"
2	kamer /ˈka:.mər/ "room"	kameel /ka.ˈme:l/ "camel"	kaag /ka:x/ "(name of lake)"
3	kaping /ˈka:.pɪŋ/ "hijacking"	kapoen /ka.ˈpu:n/ "capon"	kaap /ka:p/ "cape"
4	kapo /ˈka:.po/ "kapo"	kapot /ka.ˈpɔt/ "broken"	kaal /ka:l/ "bald"
5	kabel /ˈka:.bəl/ "cable"	katrol /ka.ˈtrɔl/ "pulley"	kaart /ka:rt/ "card"
6	kater /ˈka:.tər/ "male cat"	katern /ka.ˈtɛrn/ "section"	kaant /ka:nt/ "eats (coll.)"
7	kajak /ˈka:.jɑk/ "kayak"	cadans /ka.ˈdɑns/ "cadence"	kaan /ka:n/ "pork crackling"
8	kaki /ˈka:.ki/ "khaki"	cacao /ka.ˈkau/ "cocoa"	kaapt /ka:pt/ "hijacks"
9	kaken /ˈka:.kən/ "jaws"	katoen /ka.ˈtun/ "cotton"	kaak /ka:k/ "jaw"
10	kano /ˈka:.no/ "canoe"	kado /ka.ˈdo/ "gift"	kaars /ka:rs/ "candle"
11	kader /ˈka:.dər/ "frame"	kadet /ka.ˈdɛt/ "cadet"	kaatst /ka:tst/ "bounces"
12	kavel /ˈka:.vəl/ "plot"	cafe /ka.ˈfe:/ "café"	kaats /ka:ts/ "bounce"

Table S2. List of exposure words (in Dutch /IPA/ "English") used in the 'generalization' version of Experiment 1. SW = strong-weak; WS = weak-strong.

Experiment 1B

	SW	WS	control
1	visum /ˈvi.zʏm/ "visa"	boeket /bu.ˈkɛt/ "bouquet"	ring /rɪŋ/ "ring"
2	viering /ˈvi.rɪŋ/ "celebration"	rozijn /ro.ˈzɛɪn/ "raisin"	fee /fe:/ "fairy"
3	bonus /ˈbo.nʏs/ "bonus"	konijn /ko.ˈnɛɪn/ "rabbit"	jak /jɑk/ "yak"
4	baby's /ˈbe.bis/ "babies"	seizoen /sɛɪ.ˈzun/ "season"	meel /me:l/ "flour"
5	kiwi's /ˈki.ʋis/ "kiwis"	giraf /ʒi.ˈrɑf/ "giraffe"	nar /nɑr/ "fool"
6	donut /ˈdo.nʏt/ "donut"	piloot /pi.ˈlo:t/ "pilot"	nut /nʏt/ "utility"
7	koning /ˈko.nɪŋ/ "king"	boerin /bu.ˈrɪn/ "farmer (f.)"	pil /pɪl/ "pill"
8	sesam /ˈse:.zɑm/ "sesame"	chinees /ʃi.ˈne:s/ "Chinese"	poen /pun/ "money (coll.)"
9	sonar /ˈso.nɑr/ "sonar"	pakket /pɑ.ˈkɛt/ "package"	pot /pɔt/ "jar"
10	foto's /ˈfo:.tos/ "photos"	hobo's /ɦo.ˈbo:s/ "oboes"	dans /dɑns/ "dance"
11	lotus /ˈlo.tʏs/ "lotus"	pupil /py.ˈpɪl/ "pupil"	trol /trɔl/ "troll"
12	dosis /ˈdo.zɪs/ "dose"	siroop /si.ˈro:p/ "syrup"	zoen /zun/ "kiss"
